# Hyperprolinemia type I caused by homozygous p.T466M mutation in *PRODH*

**DOI:** 10.1038/s41439-021-00159-5

**Published:** 2021-07-20

**Authors:** Rina Hama, Jun Kido, Keishin Sugawara, Toshiro Nakamura, Kimitoshi Nakamura

**Affiliations:** 1 Department of Pediatrics, Kumamoto University Hospital, Kumamoto, Japan; 2 Department of Pediatrics, Graduate School of Medical Sciences, Faculty of Life Sciences, Kumamoto University, Kumamoto, Japan; 3 Department of Pediatrics, Kumamoto Chuo Hospital, Kumamoto, Japan

**Keywords:** Calcium and phosphate metabolic disorders, Autism spectrum disorders

## Abstract

Hyperprolinemia type I (HPI) is an autosomal recessive metabolic disorder caused by defects in proline oxidase. We herein describe a case of a patient with HPI and harboring the NM_016335.4 (PRODH_v001):c.1397 C > T (p.T466M) mutation and polymorphisms in the *PRODH* gene, as detected by plasma amino acid analysis and Sanger sequencing. The patient presented with short stature, carbohydrate-rich dietary preferences, and mild intellectual disability that was suggestive of a neurodevelopmental or learning disorder.

Hyperprolinemia type I (HPI) is an autosomal recessive metabolic disorder caused by defects in proline oxidase (POX, EC: 1.5.99.8), also called proline dehydrogenase (PRODH). The *PRODH* gene is located on chromosome 22 (22q11.21) and encodes the POX enzyme, which converts proline to pyrroline-5-carboxylate (P5C) in mitochondria.

The clinical phenotype of HPI has not been clearly characterized. Patients with HPI may present with seizures, intellectual disability, language delay, autism spectrum disorder (ASD), schizophrenia, and/or bipolar disorder. Conversely, others are asymptomatic^1–3^. Very few case reports of patients with HPI have been reported worldwide, with only two described in Japan^4,5^.

An 8-year-old boy was referred to our institution for further investigation of the cause of his short stature and suspected hypoglycemia. He was the third child of healthy nonconsanguineous parents. He was born without complications at 40 weeks of gestation, with a length of 50 cm (+0.3 SD) and a weight of 2932 g (−0.9 SD). He was able to walk independently at the age of 1 year and 2 months; speech milestones were reached at the age of 2 years. He had certain food preferences; for example, although the amount of food he ingested was normal, he tended to prefer carbohydrate-rich foods such as boiled rice and disliked a protein-rich diet including meat and dairy. He had no significant past medical history; however, an incidental finding of short stature (89.7 cm [−2.0 SD]) was noted during a routine medical examination at 3 years and 6 months. The trend persisted, and at the age of 5 years, low fasting blood insulin-like growth factor-1 levels (52 ng/mL) were detected, suggesting growth hormone (GH) deficiency. He underwent further investigation to evaluate hypothalamic-pituitary function; clonidine stimulation, arginine infusion, insulin-thyrotropin-releasing hormone/luteinizing hormone-releasing hormone, L-dopa, GH-releasing peptide-2, and Fishberg concentration tests were all normal. At the age of 7 years, he developed frequent vomiting, and was a hypoglycemic attack was suspected. Nevertheless, he presented fasting normoglycemia (91 mg/dL) at admission, and his adrenal function test was normal. Acylcarnitine profile analysis revealed no abnormalities. However, plasma amino acid analysis detected high proline levels (530 μmol/L; reference: 78 − 273 μmol/L) (Table 1A). An intelligence assessment using the Wechsler Intelligence Scale for Children-Fourth Edition (WISC-IV) at the age of 6 years and 3 months revealed the following: a score of 83 in Full Scale IQ (FSIQ), 80 in Verbal Comprehension Index (VCI), 115 in Perceptual Reasoning Index (PRI), 68 in Working Memory Index (WMI), and 78 in Processing Speed Index (PSI). These characteristics suggest a tendency toward neurodevelopmental disorders, including autism, attention-deficit hyperactivity disorder, and learning disorders.

**Table 1A:** Plasma amino acid and *PRODH* genetic analysis in our patient and literature review. A. Analysis of plasma amino acids.

Amino acid	Reference (μmol/L)	Age: 8 years	Age: 10 years 2 months
Hydroxyproline	≤21.6	11.3	28.0
Threonine	66.5–188.9	81.8	101.2
Serine	72.4–164.5	106.9	122.3
Asparagine	44.7–96.8	43.5	60.4
Glutamic acid	12.6–62.5	28.0	18.3
Glutamine	422.1–703.8	494.6	573.0
Proline	77.8–272.7	530.2	624.5
Glycine	151.0–351.0	203.4	184.9
Alanine	208.7–522.7	292.7	390.3
Citrulline	17.1–42.6	29.2	21.2
Valine	147.8–307.0	130.5	218.2
Cystine	13.7–28.3	9.1	11.9
Methionine	18.9–40.5	16.0	28.6
Isoleucine	43.0–112.8	34.9	65.5
Leucine	76.6–171.3	67.7	128.2
Tyrosine	40.4–90.3	57.9	91.6
Phenylalanine	42.6–75.7	60.0	90.6
Histidine	59.0–92.0	68.0	78.4
Tryptophan	37.0–74.9	59.1	71.1
Ornithine	31.3–104.7	33.1	53.5
Lysine	108.7–242.2	94.2	149.6
Arginine	53.6–133.6	61.7	101.4
Total AA	2068.2–3510.3	2587.9	3248.7
NEAA	1381.6–2379.4	1966.7	2317.3
EAA	660.0–1222.3	612.2	931.4
BCAA	265.8–579.1	233.1	411,9
EAA/NEAA	0.40–0.63	0.31	0.4
BCAA/Total AA	0.11–0.18	0.09	0.13
Fisher ratio	2.43–4.40	1.98	2.26

AA: amino acids, BCAA: branched-chain amino acids, EAA: essential amino acids, NEAA; non-essential amino acids.

At the age of 8 years, he ate fish but had little pork and meat. At the age of 10 years and 2 months, he had consumed more meat and pork than before.

Upon referral to our team, we further investigated the patient’s developmental history. His mother reported that he could not keep up with regular class studies in primary school or perform detailed work. He had difficulty in reading and writing Kanji. WISC-IV intelligence assessment at 9 years and 9 months was 73, 86, 91, 63 and 67 for FSIQ, VCI, PRI, WMI, and PSI, respectively, indicating mild intellectual disability. Neurological examination revealed slightly poor coordination, but there was no history of impulsive behavior. Brain magnetic resonance imaging (MRI) (Fig. 1A) and magnetic resonance spectroscopy revealed nonspecific findings, and his electroencephalogram was unremarkable. However, Sanger sequencing detected the homozygous mutation c.1397 C > T (p.T466M) in the *PRODH* gene as well as some homozygous variants (Fig. 1B and Table 1B), confirming the diagnosis of HPI. No pathogenic variants were detected in *ALDH4A1*, which encodes P5C dehydrogenase (EC 1.2.1.88) (data not shown), defects of which cause hyperprolinemia type II. At the age of 10 years and 3 months, the patient was doing well in a supported education class and did not require medication. Written informed consent was obtained from the patient’s parents for this report. This study was approved by the Institutional Ethics Committee of the Faculty of Life Science, Kumamoto University.

**Fig. 1:**
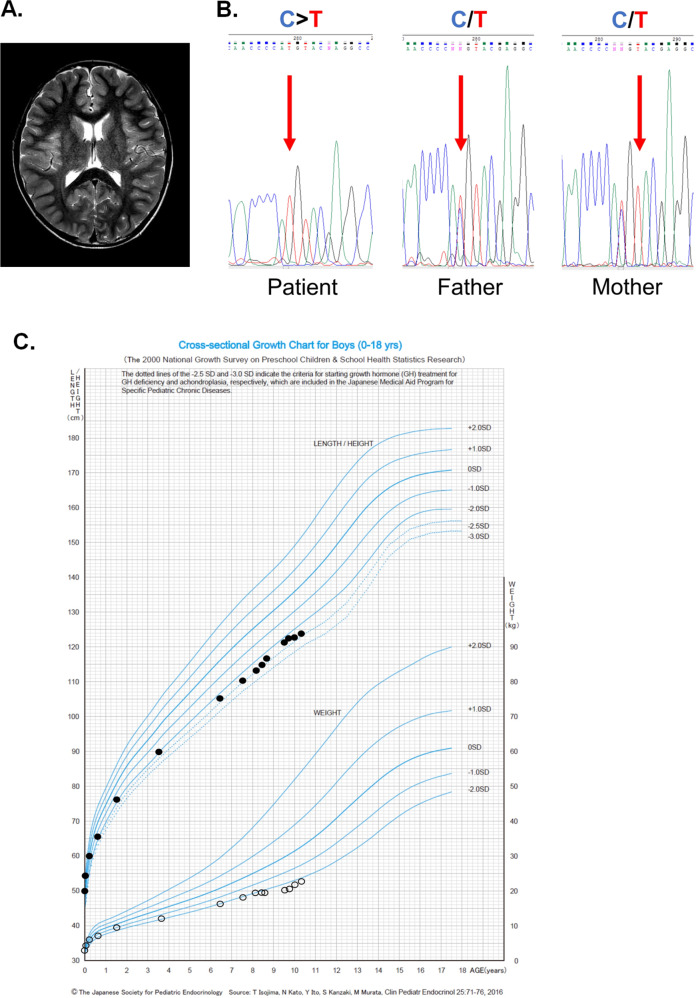
Clinical manifestations in our case. **A** Brain MRI (T2-weighted image). **B** Sanger sequencing. The patient’s father and mother carried a heterozygous mutation of c.1397 C > T (p.T466M) in the *PRODH* gene. **C** The growth curve of the patient from birth. Filled circles, height measurements; open circles, weight measurements. These circles are superimposed onto the Cross-sectional Growth Chart for Boys (0–18 y) provided by the 2000 National Growth Survey on Preschool Children & School Health Statistics Research. The height of our patient was −2.0 SD of the mean height for his age among Japanese male children. The dotted lines at −2.5 SD and −3.0 SD of height indicate the criteria for starting growth hormone (GH) treatment for GH insufficiency and achondroplasia. SD: standard deviation.

**Table 1B:** *PRODH* genetic variants, detected in our patient.

RefSNP ID	Nucleic acid	Amino acid	Location	Allele frequency in controls (%)	ClinVar	PolyPhen-2 (Score)	Human Splicing Finder
rs2008720	c.56C>A	p.P19Q	Exon 2	83.68^a,b^, 29^c^	−	Benign	−
rs4819756	c.553T>C	p.W185R	Exon 5	96.72^a,b^, 48^c^, 33.14^d^	−	Benign	−
rs1808320	c.991T>C	p.L331=	Exon 9	83.74^a,b^	−	−	probably no impact on splicing
rs1076466	c.1105–14C>T	−	Intron 10	80.89^a,b^, 39^c^	−	−	probably no impact on splicing
rs2870984	c.1397C>T	p.T466M	Exon 12	2.36^a,b^, 1.40^d^	Conflicting interpretations of pathogenicity	Possibly damaging (0.943)	−
rs2870983	c.1414G>A	p.A472T	Exon 12	3.96^a,b^, 5.31^d^, 10.29^e^, 7.02^f^	−	Benign	−
rs455072	c.1515T>C	p.F505=	Exon 13	92.06^a,b^	−	−	probably no impact on splicing
rs450046	c.1562G>A	p.R521Q	Exon 14	96.11^a,b^, 5.37^d^	−	Possibly damaging (0.507)	−
rs372055	c.1741C>T	p.L581=	Exon 15	79.59^a,b^, 28.49^d^	Benign	−	probably no impact on splicing

The variants which detected in our patient were all of homozygous. The c.1562 G > A (p.R521Q) was found in family members including the patient’s father, mother, brother, and sister as homozygous. They did not present with hyperprolinemia.

^a^Tadaka S, Saigusa D, Motoike IN, Inoue J, Aoki Y, Shirota M et al. jMorp: Japanese multi omics reference panel. Nucleic Acids Res 2018; 46: D551-D557.

^b^Tadaka S, Katsuoka F, Ueki M, Kojima K, Makino S, Saito S et al. 3.5KJPNv2, An allele frequency panel of 3,552 Japanese Individuals including the X chromosome. Hum Genome Var. 2019; 6: 28.

^c^Williams HJ, Williams N, Spurlock G, Norton N, Zammit S, Kirov G et al. Detailed analysis of PRODH and PsPRODH reveals no association with schizophrenia. Am J Med Genet B 2003; 120: 42–46.

^d^Ota VK, Bellucco FT, Gadelha A, Santoro ML, Noto C, Christofolini DM et al. PRODH polymorphisms, cortical volumes and thickness in schizophrenia. PLoS ONE 2014; 9: e87686.

^e^Jacquet H, Raux G, Thibaut F, Hecketsweiler B, Houy E, Demilly C et al. PRODH mutations and hyperprolinemia in a subset of schizophrenic patients. Hum Mol Genet 2002; 11: 2243–2249.

^f^Jacquet H, Demily C, Houy E, Hecketsweiler B, Raux G, Lerond J et al. Hyperprolinemia is a risk factor for schizoaffective disorder. Mol Psychiatry 2005; 10: 479–485.

In Table 1C, we summarize nine cases, including ours, of the POX p.T466M variant^1,6,7^. The *PRODH* gene, which is located in the 22q11 chromosomal region, is hemideleted in 22q11 deletion syndrome, also known as velo-cardio-facial syndrome (VCFS)^8^. VCFS shares some clinical features with HPI.

**Table 1C:** Reported cases of HPI with p.T466M variant.

Patient No.	1	2	3	4	5	6	7	8	9
Sex (age at diagnosis)	M (9)	M (6.5)	M (7)	F (13)	M (3)	M (3)	M (9)	M (13)	M
Autism	+−	+	+	+	−	−	−	−	N.A
Seizure	−	Febrile	−	+	+	+	−	+	N.A
Psychomotor delay	−	+	+	+	−	−	−	−	N.A
Hypotonia	−	−	+	−	−	−	−	−	N.A
Unbalanced diet	+	N.A	N.A	N.A	N.A	N.A	N.A	N.A	N.A
Language disorder	−	Few words	Short sentence	−	−	−	+	−	N.A
Intellectual disability	+−	+	+	+	+	+	−	−	+
Aggressiveness	−	+	−	−	−	−	−	−	N.A
Plama proline level (μmol/L)	530–625	930–1,000	595–715	637–1,667	1,200	414–804	679	605	N.A
MRI	Normal	CC enlargement	Mild CC enlargement	Normal	Normal	Abnormal	Normal	Normal	N.A
22q11 microdeletion	−	+	−	N.A	N.A	N.A	N.A	N.A	+
Variants	**T466M**/**T466M** + R521Q/R521Q	**T466M** + W185R	**T466M**/**T466M** + R453C	**T466M**/R453C +R431H	**T466M**/**T466M** + R453C/R453C	**T466M** + R453C/Q19P	**T466M** + R453C+W185R+Q19P+P30S/R431H	**T466M** + Q19P+W185R/R431H	**T466M**
Reference	This study	Afenjar et al. (2007)		Guilmatre et al. (2010)					Chérot et al. (2018)

CC: corpus callosum, N.A: not available

A combination of mutations and polymorphisms in the *PRODH* gene cause dysregulation of POX enzyme activity and may lead to a variety of phenotypes in HPI, including neuronal function disorders. The proline metabolic pathway links cellular proline levels with glutamate and glutamine levels in neurons, and POX has been proposed to play a regulatory role in glutamatergic neurotransmission by affecting the cellular concentration of glutamate^9^. Moreover, proline is thought to induce oxidative stress in the rat brain^10,11^. Hyperprolinemia induces significant oxidative damage to proteins, lipids, and DNA^12^, decreases the activities of antioxidant enzymes, and induces lipid peroxidation in the blood of rats^13^.

Mouse models of POX deficiency, which is also present in individuals with schizophrenia, exhibit increases in neurotransmitter release at glutamatergic synapses as well as deficits in associative learning and response to psychomimetic drugs^14^. Furthermore, hyperprolinemia has been suggested to be a risk factor for schizophrenia^14,15^, and polymorphisms in *PRODH*, such as rs372055, which this case harbored, are thought to correlate with schizophrenia^16^.

Some pharmacological, biochemical, and behavioral studies have suggested the involvement of the glutamatergic system in ASD pathology^17^, and ASD has been considered a common clinical manifestation of HPI^1,2,6^. Poor social, adaptive, and academic skills are often evident in patients with HPI^3^. Our patient displayed a complex learning disorder, including deficits in reading, writing, and math, and required specific educational training. Although this case and other reports suggest a correlation between intelligence and plasma proline levels^6,8^, further studies are required for confirmation.

Bender et al. proposed the following classification of *PRODH* mutations based on POX activity reduction: mild (<30%), moderate (30–70%), and severe (>70%)^18^. POX uses flavin adenine dinucleotide (FAD) as a cofactor. The homozygous c.1397 C > T (p.T466M) mutation results in reduced enzyme activity to less than 20% of the control^18^. T466 in POX interacts with the adenine moiety of FAD to stabilize noncovalent binding of the cofactor to the POX apoenzyme, and the T466M mutation alters the affinity of the POX apoenzyme for FAD.

Genotype-phenotype correlations in HPI have been suggested^2,6^. For example, Rosa et al.^3^ reported two patients with the same *PRODH* genotype and the same range of plasma proline levels (376 and 493 μmol/L); these patients presented with a similar neurobehavioral profile, including aggressiveness and sexual disinhibition. Our patient first presented with short stature and mild hyperprolinemia but without obvious intellectual disability, though developmental delay became more noticeable with age. Alexandra et al.^1^ described a patient with the p.T466M variant who presented with clinical manifestations and blood proline levels similar to those in our case. Although p.T466M is predicted to be damaging (0.943) in Polyphen 2 (http://genetics.bwh.harvard.edu/pph2/), ClinVar (http://www.ncbi.nlm.nih.gov/clinvar) provides conflicting interpretations of its pathogenicity (Table 1B). Therefore, the clinical impact of p.T466M remains unclear. Although short stature has not been reported as a clinical manifestation of HPI, our case showed short stature persisting from infancy (Fig. 1C). Harries et al.^19^ reported persistent short stature until the age of 27 months in a patient with HPI receiving a low-proline diet. Moreover, van de Ven et al*.*^20^ described a patient with HPI presenting with a variable eating behavior pattern that is consistent with that of our patient. Indeed, our patient preferred a carbohydrate-rich diet and disliked protein-rich foods. The manifestations of short stature and food preference may be derived from HPI, but more case reports are needed to clarify this association.

In conclusion, we encountered a case of HPI that was first detected through plasma amino acid analysis performed during the detailed evaluation of short stature in a child. Mild intellectual disability, mild learning disorders, autism tendencies, and attention-deficit hyperactivity disorder (ADHD) tendencies are considered phenotypes related to HPI. The patient’s unique dietary habit is also thought to be one of the phenotypes of HPI, and blood proline levels vary depending on the dietary content. As very few cases of HPI have been reported to date, other patients may display as-yet-unidentified phenotypes. The accumulation of more cases is essential to further our understanding of the clinical characteristics of HPI.

## Data Availability

The relevant data from this Data Report are hosted at the Human Genome Variation Database at 10.6084/m9.figshare.hgv.3045 10.6084/m9.figshare.hgv.3048 10.6084/m9.figshare.hgv.3051 10.6084/m9.figshare.hgv.3054 10.6084/m9.figshare.hgv.3057 10.6084/m9.figshare.hgv.3060 10.6084/m9.figshare.hgv.3063 10.6084/m9.figshare.hgv.3066 10.6084/m9.figshare.hgv.3069
